# The status of industrialization and development of exosomes as a drug delivery system: A review

**DOI:** 10.3389/fphar.2022.961127

**Published:** 2022-10-11

**Authors:** Yi Yin, Xing Han, Cheng Li, Tonghui Sun, Kailin Li, Xionghao Liu, Mujun Liu

**Affiliations:** ^1^ Xiangya School of Medicine, Central South University, Changsha, Hunan, China; ^2^ School of Basic Medical Science, Central South University, Changsha, Hunan, China; ^3^ Center for Medical Genetics and Hunan Key Laboratory of Medical Genetics, School of Life Sciences, Central South University, Hunan, China; ^4^ Department of Cell Biology, School of Life Sciences, Central South University, Changsha, Hunan, China

**Keywords:** exosomes, drug delivery system, loading, targeting, therapy

## Abstract

Exosomes, as natural biomolecular carriers produced by cells, have the potential and advantage of delivering drugs to target organs or cells *in vivo*. The steps to improve exosomes as a drug delivery system can be divided into three steps:large-scale preparation of exosomes, loading of drugs and targeted delivery of exosomes. Based on the existing production process and technology, there is still much room for improvement. This review highlights the research progress in three aspects and proposes new technologies and innovative approaches to improve the efficiency of exosome delivery.

## 1 Introduction

**FIGURE 1:**
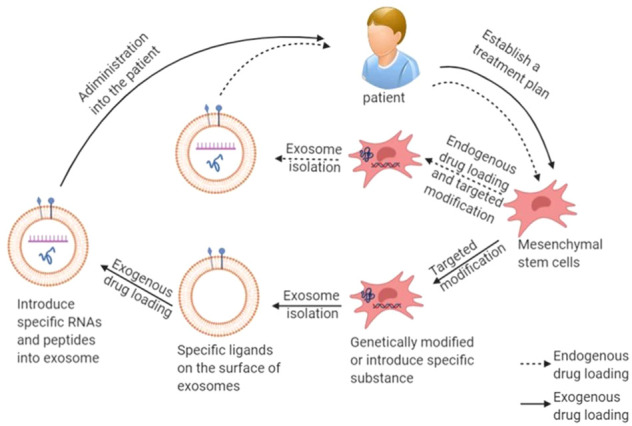
Schematic diagram of production and delivery of exosomal drug: mesenchymal stem cells are obtained from the patient’s own body to produce exosomes, and then endogenous or exogenous drug is loaded, and finally injected into the patient.

In recent years, various nano-drug formulations, such as artificially synthesized liposomes, polydopamine, nanocapsules, dendrimers, and micelles (Selvamuthukumar et al., 2012; Cheng et al., 2019), have been developed to improve the therapeutic efficacy. However, the traditional delivery system cannot be used wildly because its cytotoxicity, poor stability, immunogenicity, and poor targeting, which are unfavorable for clinical applications (Zhang et al., 2016). Meanwhile, the new drug delivery system-exosome can better avoid the above problems. Because of the biological origin, exosomes have low immunogenicity and cytotoxicity, which can effectively avoid the degradation by various enzymes in body fluids, so exosomes can stably exist in the body. The size of exosomes ranges from 30 to 150 nm and their lipid bilayer structure makes them highly permeable and easily absorbed by cells. Furthermore, they can even cross the blood-brain barrier and deliver the targeted drug to the brain (Alvarez-Erviti et al., 2011). At the same time, exosomes can participate in cell-to-cell communication in addition, exosomes can be loaded with proteins, RNA, and other small molecule drugs for targeted therapy, such as exosomes carrying catalase, RAD51-siRNA, miR-214, long non-coding RNA (LncRNA)-H19, doxorubicin is used in the treatment of Parkinson’s disease, prostate cancer, breast cancer and other diseases (Wu et al., 2017; Tai et al., 2018) (Figure 1). Conclusively, exosome is an ideal drug-delivery tool. Nonetheless, as a drug delivery system, exosomes still have many deficiencies, such as low yield, challenging production, limited drug load, affected activity of some drugs, and low targeting efficiency in the clinic. Therefore, this review will summarize the up-to-date research from production, loading and targeting of exosomes with purpose of providing directions for clinical application of exosomes.

## 2 Production process of exosomes

### 2.1 Production status of exosomes

Various types of cells can produce exosomes, such as macrophages, dendritic cells, t, B, natural killer cells (nks), epithelial cells, endothelial progenitor cells, fibroblasts, tumor cells and stem cells (Pandian et al., 2022). Exosomes from different sources can produce effects against different diseases. For example, macrophages produce exosomes that contribute to cellular signaling in many pathologies, especially in cardiovascular diseases (Phu et al., 2022).

Red blood cells (RBCs), are the most studied cells as biological carriers. The advantage is that RBCs can effectively target the reticuloendothelial system. And the release of the drug and the maintenance of the plasma drug concentration can be controlled (Sherif et al., 2019). However, cells as drug transport carriers still face many problems, such as uncertain cell differentiation, cell embolism, infection, and difficulties in production and storage. They are not suitable for targeting other organs and tissues, and biocompatibility and immunogenicity also are one of the main limitations of these vectors.

Initially, exosomes were isolated from biological fluids such as blood and milk. However, the main disadvantages of this method are that the number of exosomes produced is small and easy to be contaminated. In recent years, exosomes from embryonic stem cells (ESCs), induced pluripotent stem cells (iPSCs) and mesenchymal stem cells (MSCs) have received great attention. MSCs can produce exosomes on a large scale (Yeo et al., 2013) Mesenchymal stem cells are multifunctional stem cells present in a variety of human tissues, commonly found in the spinal cord, placenta, adipose and other tissues. Many studies have shown that exosomes, as a product of MSCs, can not only regulate the expression of components, but also mediate the occurrence and development of cancer through a variety of signaling pathways (Zhou et al., 2021). Because of their low immunogenicity, multidirectional differentiation ability, therapeutic benefits, and specific homing ability, MSC-based drug delivery systems have become a current research hotspot. MSC-secreted exosomes (MSC-EXOs) successfully inherit the advantages of MSCs and overcome the major problems faced by cells as drug delivery carriers. For example, nucleic acid drugs are unstable in biological liquids, which makes them difficult to deliver. However, studies have shown that exosomes isolated from MSCs can be effectively delivered as biological carriers. Also, the treatment of graft-versus-host disease patients with MSC derived exosomes showed that repeated injection was well tolerated and did not cause cytotoxicity (Kalluri and Lebleu, 2020). A good manufacturing practice-grade standard protocol for the production of exosomes from mesenchymal stem cells has been widely discussed (Pachler et al., 2017; Mendt et al., 2018; Rohde et al., 2019; Witwer et al., 2019). Moreover, due to the critical discovery that mesenchymal stem cells play a role through paracrine, more and more studies are exploring the potential function of exosomes derived from mesenchymal stem cells. Mesenchymal stem cells can express and secrete growth factors, cytokines, signal peptides, and other substances by paracrine and then affect cell apoptosis, differentiation, and other life activities. In summary, MSCs have become a suitable cell source for the industrial production of exosomes. However, the production process and purification method of exosomes are still a major challenge hindering the industrial production of exosomes, and the current production process is not standardized (Elsharkasy et al., 2020).

### 2.2 Improvements in the culture process of exosomes

Exosomes are secretions of cells. Thus, not changing the cell phenotype is the focus of the mass production process of exosomes. At present, the large-scale production of exosomes is mainly long-term production in large culture flasks. This 2D culture method will be limited by the loss of cell cloning and differentiation ability and limited expansion ability in the long-term passage (Mckee and Chaudhry, 2017). Compared with the traditional culture method, the conditioned medium produced by the hollow fiber bioreactor culture can obtain about 40 times more exosomes per milliliter (Watson et al., 2016). Additionally, due to the accumulation of exosomes and smaller space, the concentration of exosomes increases, making it easier to obtain samples with high concentration and purity. Studies have shown that the phenotypes of cells and exosomes have not changed when using bioreactors to expand MSCs(Mendt et al., 2018).

Traditional 2D cell culture changes the shape of cells, leading to changes in the cytoskeleton, which in turn affects the gene expression of cells (Wang et al., 2017b). To avoid these problems and increase production, Kim (Kim M. et al., 2018) found that the 3D hanging drop sphere (3D-HD) and the 3D poly (2-hydroxyethyl methacrylate) (poly-HEMA) spheroids (3D-PH) can produce more exosomes from MSCs than the traditional 2D culture. Similarly, Haraszti (Haraszti et al., 2018) still found that MSCs derived from umbilical cord produced 20 times more exosomes produced by 3D culture-differential ultracentrifugation than 2D culture; The exosomes produced by combining 3D culture with tangential flow membrane filtration technology are seven times higher than those produced by 3D culture. Moreover, compared with exosomes produced by 2D culture, exosomes produced by 3D culture deliver siRNA to neurons with seven times higher efficiency (Haraszti et al., 2018). In summary, the yield of 3D culture has been significantly improved, and the cell phenotype in 3D cell culture is more stable, which is more suitable for developing drug delivery systems (Souza et al., 2018). Nevertheless, there are still two limitations in the 3D cell culture at least. First, the 3D-HD culture method requires manual enlargement of the hanging objects, and the operation is not simple. Second, the production efficiency of exosomes in the 3D-PH culture method is affected by the size of the sphere, and the production stability is poor.

### 2.3 Improve the purification process of exosomes

In 2016, a survey on the research methods of extracellular vesicles showed that most researchers use conditioned medium to produce extracellular vesicles and ultracentrifugation to isolate extracellular vesicles (Gardiner et al., 2016). However, ultracentrifuges are expensive (an ultracentrifuge is required), time-consuming, require a large number of samples, and lead to the aggregation of exosomes (Linares et al., 2015). Even worse, exosomes can be damaged during operation (Gupta et al., 2018). MISEV2018 also pointed out that pure ultracentrifugation lacks specificity (Théry et al., 2018). Researchers expect to obtain sufficient clinical-grade exosomes by improving methods of purification. Gupta proposed one-step sucrose cushion ultracentrifugation (Gupta et al., 2018). After adding the appropriate amount of sucrose at the bottom of the test tube and accordingly modifying the process of ultracentrifugation, the number of exosomes obtained by this method is about 2–3 times that obtained by ultracentrifugation, and the surface marker protein expression of exosomes is higher. Shengming Dai and his colleagues (Dai et al., 2008) successfully isolated exosomes from ascites of patients with colorectal cancer by sucrose cushion ultracentrifugation in the first phase of a clinical trial, which is used in the treatment of patients with advanced solid tumors and successfully induced apoptosis of solid tumor cells. To overcome the aggregation problem caused by ultracentrifugation, researchers considered a method with first concentrating exosomes in a liquid cushion and subsequently resolving them using density gradient ultracentrifugation and achieved remarkable results (Li et al., 2018; Onódi et al., 2018; Duong et al., 2019). Mitja L (Heinemann et al., 2014) and other researchers designed a three-step filtration protocol for exosome isolation. First, dead-end pre-filtration was performed to remove cells and cell debris; then, tangential flow filtration was performed to remove the free protein, and the sample was initially concentrated; finally, the purified exosomes were obtained by track etch filtration. Compared with ultracentrifugation, this method has the advantages of high speed, high expansibility, and specificity, which is very helpful for the clinical application of exosome isolation.

Due to the low specificity of the separation method based on the particle size and density, resulting in the low purity, some researchers turn their attention to the structure of the exosome’s surface. Immunomagnetic beads are spherical magnetic particles with monoclonal antibodies against exosome surface proteins, which can specifically bind to exosomes. Mizutani K’s team successfully isolated cancer cell exosomes from the plasma of patients with prostate cancer using CD9 antibody and anti-prostate specific membrane protein antigen modified immunomagnetic beads (Mizutani et al., 2014). In 2020, Poellmann MJ (Poellmann et al., 2020) designed a dual-layer dendrimer configuration to capture exosomes. Due to the multivalent binding effect of the configuration and the increase of short-range adhesion mediated by the configuration, the separation effect of exosomes is significantly improved. Although the purity of exosomes isolated by the immunomagnetic beads method is higher than that by ultracentrifugation, the immunomagnetic beads method is not suitable for large-scale purification due to the long time required for antigen-antibody binding. If the exosomes exist in the eluent for a long time, whether their biological activities will change remains to be verified.

Ibsen SD. et al. (Ibsen et al., 2017) [33] designed a chip device. Firstly, the exosomes were separated rapidly by electrostatic force, and then the captured exosomes were detected by immunofluorescence technology, which not only isolated a large number of exosomes but also had high specificity. Although the purity and efficiency of nanochips based on microfluidic technology have been greatly improved, due to the cumbersome design and expensive equipment, it is still in the research stage.

## 3 Loading capacity of exosomes

In 2011, Alvarez-Erviti et al. (Alvarez-Erviti et al., 2011) first proved the hypothesis of exosomes as drug carriers. They injected the exosomes loaded with lamp2b protein and siRNA into mice, and the result showed that the exosomes could transport siRNA through the blood-brain barrier and inhibit the expression of Alzheimer’s related gene BACE-1(Beta-Secretase 1). However, the low loading efficiency of exosomes limits the clinical application of exosomes as drug delivery systems. The drug loading modes of exosomes can be divided into exogenous and endogenous. In recent years, many researchers are exploring more efficient loading modes.

### 3.1 Exogenous drugs loading

**FIGURE 2:**
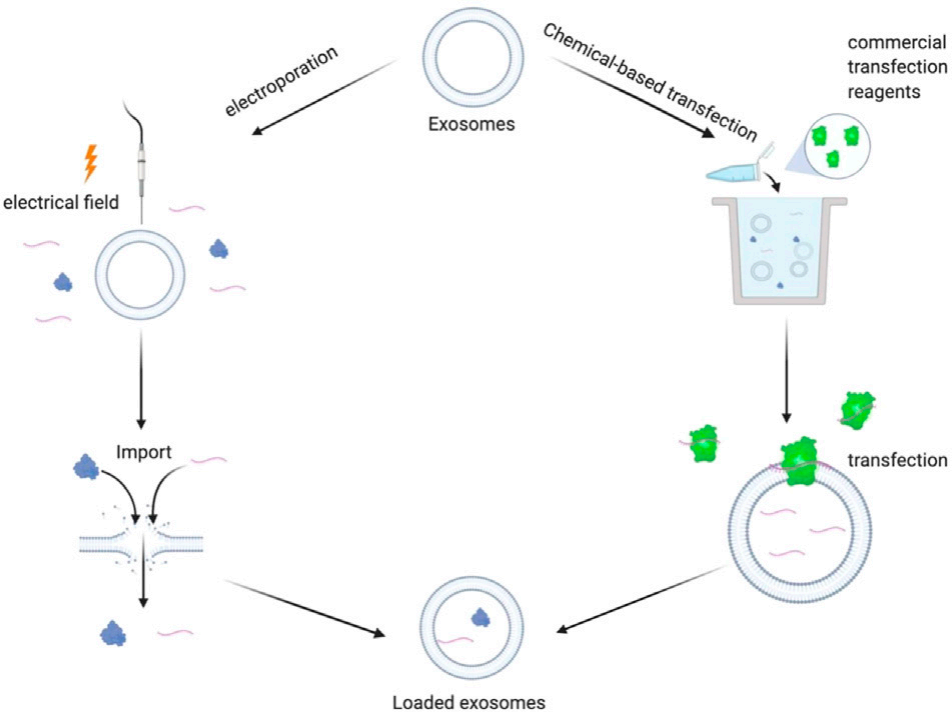
Schematic diagram of exogenous drug loading principle: electroporation method: under the action of external electric field, the phospholipid bilayer produces repairable pores, and the drug enters the exosomes through the holes. Chemical transfection: under the action of commercial transfection agent, exogenous drugs enter the exosomes.

In exogenous drug loading, the exosomes are isolated and purified from donor cells. Next, drugs are loaded directly into the purified exosomes by the action of physical and chemical factors, such as an external electric field or chemical transfection agent in a culture environment, as shown in Figure 2.

#### 3.1.1 Electroporation

Electroporation is the application of an electric field to the cell culture environment. When the electric field force gradually increases, the lipid bilayer will rearrange to form hydrophilic channels, so that small molecules can enter exosomes. When the external electric field stops, the lipid molecules will recover their original stable structure. In 2017, Wang et al. (Wang et al., 2017a) loaded siRNA and miRNA into the exosomes modified by tumor-targeting ligand AS1411 by electroporation and successfully delivered siRNA and miRNA to breast cancer tissue, significantly inhibiting the growth of tumor cells. However, the traditional electroporation method has poor ability in carrying macromolecular nucleic acids. To solve the problem, Yang et al. (Yang et al., 2020) invented a new type of cellular nanoporation biochip (CNP) in 2019. First, the donor cells were incubated in the buffer solution of plasmid DNA. After the directional current was given, the cell membrane was damaged, and the plasmid entered the cell through the nanopore along with the potential difference. After that, the cells began to repair the membrane and transcribe the plasmid DNA to mRNA. Meanwhile, exosomes were secreted to expel the foreign plasmids. Compared with the traditional electroporation technology, CNP overcomes the problem that macromolecular mRNA is challenging to be loaded into exosomes, and the exosome production with target mRNA is increased by 1,000 times.

However, in CNP technology, electroporation will lead to problems such as precipitation and inactivation of siRNA [36]. It is shown that aggregate formation decreased with increasing concentrations of EVs. This effect may be due to the capturing of multivalent ions on the negatively charged EV membrane or by changes in buffer conductivity upon adding EVs. In 2014, Hood (Hood et al., 2014) and others added 50 mM membrane stabilizer trehalose into PBS buffer solution after the electroporation process at 0.75 kV/cm. Trehalose can form a unique protective film on its surface, effectively protecting the structural integrity of biomolecules. However, trehalose will increase the viscosity of the buffer solution, hinder the drug from entering exosomes, and affect the loading efficiency. Electroporation has the advantages of safety and easy control of parameters. However, problems such as exosome aggregation and drug destruction have not been solved completely. Therefore, constructing a drug delivery system by electroporation still needs to be optimized and improved.

#### 3.1.2 Chemical-based transfection

Chemical-based transfection is a method that cultures together with the drugs with the purified exosomes. Under the action of the transfection reagents, the target drug is loaded into the exosomes. The reagent is a unique blend of cationic and neutral lipids that enables to uptake of siRNA and release of siRNA inside cells. As early as 2012, Jessica Wahlgren (Wahlgren et al., 2012) successfully loaded siRNA into plasma exosomes under the action of the Hiperfect Transfection Reagent and could deliver the exosomes to monocytes and lymphocytes. Haney et al. (Haney et al., 2015) loaded catalase into the exosomes by adding saponin, thereby increasing the drug loading rate by 18.5% compared with the traditional passive diffusion method based on the similar solubility principle. The system successfully made catalase cross the blood-brain barrier and reach the brain, thus improving the disease status of Parkinson’s disease. However, chemical-based transfection lacks safe and effective transfection reagents, and the transfection reagents and exosomes cannot be separated, which may cause further damage to the target cells. Gregor Fuhrmann (Fuhrmann et al., 2018) cultured together exosomes, β-glucuronidase with saponin solution, then obtained exosomes loaded β-glucuronidase by purification. This method loaded β-glucuronidase into exosomes effectively without adverse effects on exosomes. Up to now, the therapeutic effect of chemical-based transfection as a drug-loaded method is still controversial. Several questions must be settled: 1. The residual transfection reagents caused cytotoxicity. 2. It is hard to determine whether the drugs after the transfer exists in the exosome or the exosome surface. 3. The most significant is that a new type of transfection agent that not only efficiently transfer the drugs but also cannot cause cytotoxicity needs to be applied.

### 3.2 Endogenous drugs loading

Endogenous drug loading refers to loading drugs into exosomes in cells. Traditional methods usually use chemical transfectants, but transfectants are usually cytotoxic. The types and doses of transfectants are subject to many restrictions. This leads to increased costs and more complicated processes, which hinders industrial production and clinical applications. However, endogenous drug loading, as the main mode of production, is still the focus of research in recent years.

#### 3.2.1 Endogenous delivery of protein and polypeptide macromolecular drugs

At present, there is a lack of an efficient separation mechanism between foreign protein and exosome vesicles, which leads to the limitation of drug loading efficiency and makes it more challenging to purify exosomes. Yim et al. (Yim et al., 2016) developed a new tool for drug delivery into exosomes, named ‘exosomes for protein loading *via* optically reversible protein-protein interactions’ (EXPLORs). The EXPLORs can efficiently deliver the protein into exosomes by binding the photoreceptor cryptochrome 2 (CRY2), a blue-light dependent phosphorylase, to a basic helix loop helix 1 (C1B1), and the binding process can be reversibly modulated by blue-light. Both ends of the CRY2-C1B1 complex can bind to CD9 and target proteins on the exosomal membrane, respectively, and bind or dissociate with blue light irradiation. The experimental results show that when the intermittent dark cycle switches between blue light and dark every minute, the load capacity of the Explorer system reaches a peak.

Wang et al. (Wang Q. et al., 2018) discovered a blocking protein domain with ARRDC1 microvesicle (ARMMS). ARMMS is an extracellular vesicle, not an exosome. However, its special structure mode also provides a new idea. The ARRDC1 protein is located on the plasma membrane, which plays a vital role in the budding process of ARMMS, and its overexpression contributes to the formation of ARMMS. Experiments proved that by fusing p53 with ARRDC1, P53 could enter ARMMS when ARMMS sprouted, after which the loading capacity was significantly increased. In that case, each extracellular vesicle contains about 540 protein molecules on average. Moreover, under physiological conditions, its biological toxicity is low, its structure is highly stable, and the protein inside is not easy to be decomposed, which dramatically improves the efficiency of drug delivery. Therefore, an ideal strategy for developing ARMMS into a mature drug delivery system is to explore further the characteristics of ARMMS as well as the possibility of reducing its development cost and production difficulty.

#### 3.2.2 Endogenous delivery of RNA drugs

There are more studies on RNA. In 2013, Shin-ichiro Ohno et al. (Ohno et al., 2013) successfully transfected the vector plasmid carrying epidermal growth factor (EGF) into the cells by using the transfection reagent FuGENE-HD and successfully expressed exosomes containing EGF. However, it has high requirements for transfection agents. Besides, the researchers found that drug loading can be effectively increased by using the fusion protein on the membrane to assist the transport. For example, the loading efficiency can be improved by fixing drug molecules on the exosome’s membrane *via* Palm, Lamp, CD63, and CD9.

Palmitoylation signal (PalmGFP, PalmtdTomato) can be combined with sulfhydryl group on cysteine through thioester bond for exosome surface protein fusion. When it is combined with bacteriophage MS2 coat protein (an RNA binding protein), it can efficiently deliver RNA into exosomes and can provide a dynamic labeling vision to observe the detailed process of its transport to target cells, which is beneficial for the follow-up research of exosomes (Lai C. P. et al., 2015).

It showed that lysosomal membrane lamp fusion protein can also improve the loading capacity of exosomes. The miR-199a-3p sequence was inserted into the intron of Lamp 2a to realize intracellular expression. The exosomes and corresponding RNA are combined with transactivated RNA (TAR) through the TAT peptide sequence. It achieves the purpose of high-efficiency loading and increases the enrichment degree of miRNA by about 65 times (Sutaria et al., 2017). By further fusing Lamp, 2b with MS2 and labeling with HA peptide, Michelle et al. (Hung and Leonard, 2016) enriched endogenous miRNA and protein into exosomes. This method was called the Targeted and Modular EV Loading (TAMEL) platform. However, further research has shown that this method will reduce the biological activity of drug molecule RNA to a certain extent. This may be due to the existence of a certain membrane protein in the produced exosomes, which mediates the exosomes into lysosomes and inactivates the miRNAs it contains. Although the specific mechanism of this method stays unclear, it still promotes the research and development of exosomes in the treatment of diseases (Hung and Leonard, 2016; Sutaria et al., 2017).

CD63, as a protein-enriched on the surface of the exosome membrane, has been attracting much attention. Kojima et al. [28] designed a unique packaging device that used miRNA combined with bioluminescent gene (nluc) as the detection substance—combining the archaeal ribosomal protein (L7Ae) with the C-terminus of CD63 on the exosome membrane and inserting the C/D RNA sequence, which can specifically bind to L7Ae into the target RNA. C/D is a particular RNA sequence with methylation modification and pseudouridine acylation. It is assisted target RNA in entering exosomes through the interaction between L7Ae and C/D and can significantly increase the exosome load.

Efficient encapsulation of exosomes can also be realized by the membrane localization of CD9. Li et al. (Li et al., 2019) fused CD9 with an RNA binding protein HuR, which can bind to the AREs sequence on miR-155 and has a high affinity for miR-155. The fused protein CD9-HuR was then co-transfected in 293T cells and efficiently introduced over-expressed miR-155 into recipient cells according to the immunofluorescence. With the help of the CRISPR/Cas9 system, the AREs sequence was inserted the downstream of the stop codon of other RNA drugs, in which way the number of target RNA entering exosomes was also increased, but it had no effect on the expression number of mature target RNA, only feasibility of increasing drug loading was confirmed.

**FIGURE 3:**
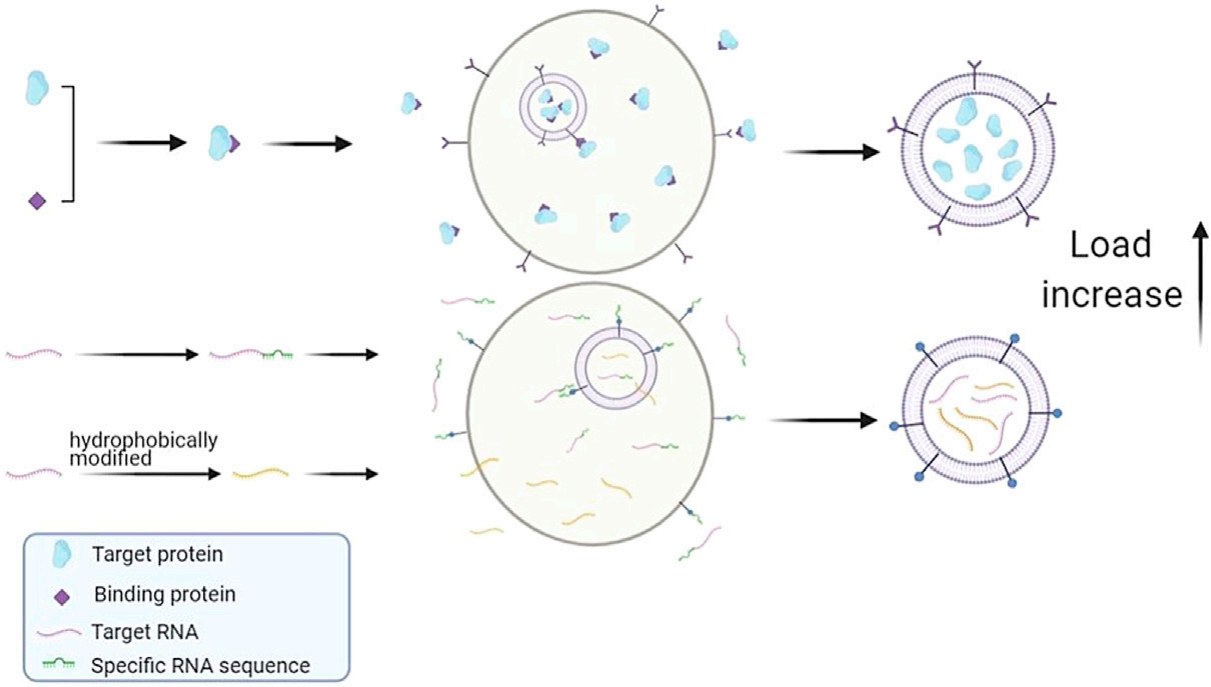
Schematic summary of improved endogenous drug loading method. Protein drugs can be fused with special binding proteins and anchored on membrane proteins to improve drug loading. Special gene sequences can be added to RNA drugs, and the loading efficiency of drugs can be improved by interacting with corresponding RNA binding proteins. Or change the hydrophobicity of RNA drugs and increase the amount of RNA entering exosomes.

The above studies confirmed that the loading value can be improved by using membrane targeting proteins or using specific sequences as mediators to help drugs enter exosomes. In addition, other studies have shown that drug loadings can be increased by changing the hydrophobicity of drugs, as shown in Figure 3. The siRNA is an asymmetric siRNA with a fully phosphorylated single-stranded tail. In the experiment conducted by Didiot et al. (Didiot et al., 2016), where the hydrophobicity of siRNA was modified, it was found that the hydrophobic-small-interfering RNA (hsiRNA) entered the exosomes efficiently and successfully targeted the primary cortical neurons of mice to functioning. This indicates that changing the hydrophobicity of drug molecules or exosome membranes by genetic engineering, physic, or chemistry is also a feasible way to improve drug loading.

In conclusion, there are still many problems to be improved. First of all, there is a lack of an efficient drug loading method that is suitable for general application. Most of the existing studies have clear limitations on the types of structures of drug molecules. Secondly, there always is a trade-off between loading capacity and process complexity/production cost. The traditional methods, such as lentivirus transfection and co-incubation, have low drug loading but also is low in cost. These methods can be produced in large quantities in a short time and can ensure drug activity and yield. Some improved exogenous methods (Wahlgren et al., 2012; Kooijmans et al., 2013; Hood et al., 2014; Haney et al., 2015; Fuhrmann et al., 2018; Yang et al., 2020), such as electroporation and ultrasound, will improve the loading efficiency several times, but they are mostly limited to RNA drugs and may damage exosome vesicles. Chemical transfection methods may contain biological toxicity that damages cells. Therefore, their suitability remains to be verified. In contrast, the improved endogenous drug loading method (Lai C. P. et al., 2015; Didiot et al., 2016; Hung and Leonard, 2016; Yim et al., 2016; Sutaria et al., 2017; Wang Q. et al., 2018; Kojima et al., 2018; Li et al., 2019) makes the loading efficiency higher through the medium, but its pertinence and limitation are also higher. Because the sorting mechanism of drug molecules in cells is complex and unclear, there is no mature technology at present. Moreover, some improved methods may also affect the molecular activity of drugs, which significantly increases the cost and difficulty of clinical application.

## 4 Targeting of exosomes

### 4.1 Targeting of exosomes and current research status

**FIGURE 4:**
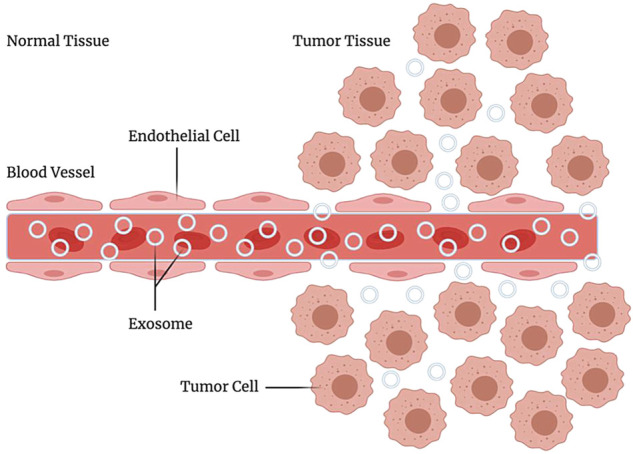
EPR effect. Enhanced permeability and retention effect, it refers to the fact that some molecules or particles of are more likely to aggregate in tumor tissue than in normal tissue.

Exosomes have many advantages as carriers of drug delivery systems, especially in targeting, which makes them an ideal drug targeting carrier. The natural targeting of exosomes is mainly from donor cells. This is closely related to the targeting molecules of exosomes. For example, liver and spleen macrophages can recruit B cell-derived exosomes through the binding of CD169 expressed by them and sialic acid on the surface of exosomes (Kooijmans S. A. et al., 2016). Homing of tumor cell-derived exosomes is beneficial for drug delivery. After reaching the tumor cells, some proteins expressed on the surface of exosomes can promote membrane interaction and fusion, thereby swallowing the drug into the cell (Tian et al., 2014). It is particularly worth mentioning that exosomes have a longer retention time at the tumor site. Thus they usually show a better tumor enrichment effect. This phenomenon is called the enhanced permeability and retention effect (enhanced permeability and retention effect, EPR) (Kalyane et al., 2019), which plays a vital role in the homing of exosomes to tumor tissues, and it is also very conducive to reflecting the targeting of tumor-derived exosomes (Figure 4). Moreover, many study reports mentioned controlled targeting of drug release (Heng, 2018). Yang et al. (Yang et al., 2015) used exosomes derived from brain endothelial cells to encapsulate adriamycin and proved that it could target brain tumors in zebrafish. Compared with adriamycin alone, adriamycin coated with exosomes has a better targeting effect on tumor cells. Due to the advantages of exosomes in targeting, many researchers have tried to use exosomes as targeted chemotherapeutic drug carriers and have achieved satisfying results (Yong et al., 2019). Yi Ba and Guoguang Ying’s group at Tianjin Medical University have made significant progress in targeted therapy of gastric cancer by encapsulating therapeutic miRNAs derived from human gastric cancer cells (Wang X. et al., 2018). These studies based on the existing targeting of exosomes have demonstrated that the use of exosomes for drug delivery is feasible.

As mentioned earlier, exosomes have a natural targeting ability, but their targeting is relatively limited, and it is challenging to meet the requirements of clinical therapeutics.

### 4.2 Targeting strategies

The limitation of exosomal targeting is reflected in two aspects, the limitation of donor cell types and the limitation of the exosomal targeting ability. In terms of donor cells, exosomes produced by only a few cells have a specific targeting capability, such as some immune cells and tumor cells. In contrast, exosomes from many other cell types are not targeted, such as exosomes derived from mesenchymal stem cells, commonly used in exosome production. Because the target of MSC exosomes is edited by housekeeping genes and exists in almost all cells, MSC exosomes have poor targeting ability to tumor cells and play an essential role in the growth and transformation of tumor cells (Lai R. C. et al., 2015). Regarding exosomes, only a few types of exosomes, such as those secreted by some immune cells, have straightforward and efficient targeting capabilities (Tian et al., 2014).

Therefore, researchers researched exosomal targeting expansion, hoping to improve the targeting of exosomes. Based on the existing research, we have carried out an innovative classification, which is related to the introduction of both direct and indirect enhancement of exosomal targeting.

#### 4.2.1 Direct enhancement of exosomal targeting

The realization of natural targeting of exosomes is mainly related to their targeting molecules. Most of the existing studies are based on the shortcomings in this aspect and try to add unique targeting molecules to the exosomal membrane by various methods to make the exosomes have artificial targeting. The following Table 1 illustrates some common targeting molecules of exosomes.

**TABLE 1:** Major exosome R & D companies.

Company	Location	Progress			
		Discovery	Preclinical	Phase 1	Website
Codiak BioSciences	America	5	2	2	https://www.mg21.com/cdak.html
The Cell Factory	Belgium	2	2	0	https://www.cell-factory.com/
United Therapeutics	America	0	0	1	https://www.unither.com/index
Avalon GloboCare	America	0	2	0	www.avalon-globocare.com
Unicyte AG	Germany	0	1	0	https://unicyte.ch/
Tavec Pharmaceuticals	Canada	0	1	0	http://www.tavecpharma.com/
Evox Therapeutics	Britain	1	0	0	https://www.evoxtherapeutics.com
Cell Tex Therapeutics	America	1	0	0	https://celltexbank.com/

**FIGURE 5:**
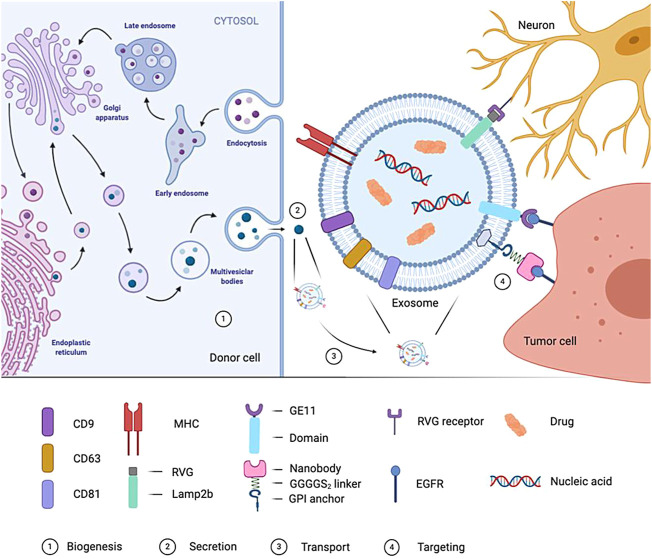
Formation of exosome and targeted process. In the genetically-modified donor cells, the biogenesis process of cell-derived exosomes is showed. Mature exosomes are released by the donor cells, transported by body fluids to reach the target cells, and interact with the target cells through specific targeting molecule.

It is the most common way to improve the target delivery efficiency of exosomes by using exosome surface molecules (Figure 5).Several strategies have been developed to target exosomes to specific cell receptors. One of the critical methods is with the help of a genetic engineer to modify exosomes and express targeted peptides on their surface. Several studies (Ohno et al., 2013; Kooijmans S. A. et al., 2016) have shown that expressing particular proteins on the surface of exosomes can target them to tumor cells expressing EGFR. Kooijmans et al. (Kooijmans S. A. et al., 2016) introduced genes encoding EGFR antibodies into the source cells of exosomes. The expressed antibody binds to the GPI anchoring signal peptide on the surface of the exosomes so that the exosomes can target and bind to tumor cells expressing EGFR. In addition, Ohno et al. [6] used genetic engineering technology to manufacture GE11 targeting peptide modified exosomes (GE11-Exos) and use GE11-Exos to target tumor suppressor miRNAs to breast cancer tissues with high expression of EGFR on the cell membrane surface. Alvarez Erviti et al. (Alvarez-Erviti et al., 2011) used virus-derived proteins to give exosome targeting ability. They modified dendritic cells through genetic engineering to express Lamp2b protein on the exosomal membrane, which can bind to rabies viral glycoprotein (RVG) polypeptide, then realizing the targeted delivery of drugs by exosomes. Table 2 illustrates the current situation of exosomes.

**TABLE 2:** Exosome drugs and progress.

Company	Indication	Cargo	Progress
Codiak BioSciences	*Cancer*	exoIL-12	Phase 1
United Therapeutics	Bronchopulmonary dysplasia	UNEX-42	Phase 1
ArunA Biomedical	Stroke	AB 126	Preclinical
Avalon GloboCare	Diabetic foot ulcer	AVA202	Preclinical
Avalon GloboCare	Pulmonary fibrosis	AVA203	Preclinical
Capricor Therapeutics	Duchenne muscular dystrophy (DMD)	CAP 2003	Preclinical
Codiak BioSciences	*Cancer*	exoSTING	Preclinical
Tavec Pharmaceuticals	cholangiocarcinorma	TVC 201	Preclinical
The Cell Factory	Bronchopulmonary dysplasia	CF-MEV-132	Preclinical
The Cell Factory	Perianal fistula	CF-MEV-107	Preclinical
Unicyte AG	Acute kidney injury	Nano-Evs	Preclinical
Sarepta Therapeutics	Duchenne muscular dystrophy (DMD)	Vyondys53 (golodirsen)	Preclinical
Sarepta Therapeutics	Duchenne muscular dystrophy (DMD)	Exondys 51 (eteplirsen)	Preclinical
Codiak BioSciences	Autoimmune disorders	exoCD-3	Discovery
Codiak BioSciences	Caner	exoVACC	Discovery

However, genetic engineering is complicated and expensive. In particular, it cannot be used for purified or patient-derived exosomes. To overcome the above shortcomings, researchers tried to modify the exosomal membrane by directly combining chemical molecules. Tian et al. (Tian et al., 2018) used the chemical method to join RGDyK cyclic peptide (which can specifically bind to the highly expressed αv integrin of tumor cells) to exosomes. The modified exosomes can contain curcumin to target the cerebral ischemia site and inhibit the inflammatory response after cerebral ischemia. In addition, the study by Kim et al. (Kim M. S. et al., 2018) demonstrated that aminoethyl ethanolamine (AA) modified exosomes can efficiently carry drugs to target lung metastatic cancer sites, and its mechanism of action is mainly about the combination between AA and sigma receptors. Related, many cancer types such as NSCLC overexpress the membrane-bound protein sigma receptor, and AA is a high-affinity ligand of the sigma receptor, so the specific combination of the two achieves targeted delivery of exosomes. Researchers have now tried to modify the exosomes containing paclitaxel with AA, and they have shown promising results in treating metastatic lung cancer. The following Table 3 illustrates some common modifications of exosomes. tLyp-1 is a short peptide which can penetrate neurofilament selectively, target tumors, and penetrate matrix. Bai J et al. (Bai et al., 2020) designed a plasmid and obtained exosomes with tLyp-1 and lamp2b fusion protein on the membrane after transfection. They used electroporation technology to load tumor drug siRNA into exosomes and improved the targeting ability of exosomes successfully.

**TABLE 3:** Common modification of exosomes.

Modification	Tumor type	Target	References
GE11 peptide	Breast cancer	EGFR	Ohno et al. (2013)
RVG-Lamp2b	Neuroblastoma	RVG-receptor	Alvarez-Erviti et al. (2011)
Aminoethylethanolamine (AA)	Lung metastatic cancer	sigma receptor	Tian et al. (2018)
RGDyK cyclic peptide	αv integrin positive breast cancer	αv integrin	Tian et al. (2014)

In addition, the exosomal targeting of tumor tissues through physical methods is a new idea to improve the exosomal targeting directly, and it has also attracted widespread attention in recent years. Qi et al. (Qi et al., 2016) used transferrin combined explicitly with the transferrin receptor on the surface of serum exosomes and incubated superparamagnetic particles with mouse serum. So that the superparamagnetic particles can be bound to transferrin on the surface of exosomes, and then they collected the exosomes through an external magnetic field and completed the drug loading. Finally, they injectedand applied a magnetic field to the tumor site to achieve the targeting. Scientists in China have successfully transformed this research into a patented production. The patent combines superparamagnetic nano-iron with exosomes and enables exosomes to target tumor tissues under the action of an external magnetic field applied by the tumor site. This research is expected to be put into clinical application in the future.

#### 4.2.2 Indirect enhancement of exosomal targeting

Methods to indirectly increase exosome targeting are closely related to the mononuclear phagocyte system MPS (mononucleophagocyteage system), whose preferential uptake of exosomes. Meanwhile, it has affected the clinical value of exosomes as targeted drug carriers (Yong et al., 2017).

Some researchers have used polyethylene glycol analogs (Kooijmans S. A. A. et al., 2016) to attach to the surface of exosomal membranes to reduce the clearance of exosomes and extend their circulation time in the body. However, PEG antibodies are found in many people, which accelerate the elimination of exosomes. Wan Z et al. (Wan et al., 2020) demonstrated that the reduction of macrophages could significantly reduce the clearance of exosomes in the liver and spleen. Other studies have shown that macrophages may take up exosomes in various ways, including Cltc-mediated endocytosis [64]. Knocking out Cltc gene can significantly reduce the uptake of exosomes by macrophages, potentially increasing exosomal accumulation in targeted tissues and improving drug delivery (Kooijmans S. A. A. et al., 2016). In addition, studies have shown that the transmembrane proteins on the surface of exosomes can affect the phagocytosis of MPS on exosomes. Kamerkar et al. (Kamerkar et al., 2017) proved that CD47 could enable exosomes to avoid the phagocytosis of MPS in relevant studies on pancreatic cancer. This phenomenon is related to the ‘do not eat me’ signal triggered by the binding of CD47-SIRPα.

### 4.3 Future perspectives for exosomal targeting

According to existing research, it is not difficult to see that the natural exosomal targeting for drug delivery is insufficient to meet the demand. In other words, there are still broad research prospects in this field. The improvement and simplification of targeted modification methods, the screening and identification of targeted molecules, and the transformation and application of related research results to clinical practice are the key directions of related research in the future, which require continuous exploration by relevant scientific researchers.

## 5 Commercial application of exosome

Exosomes are turning from academic research to the field of biotechnology and developing into clinical research. At present, nearly 50 EVs related companies have emerged that focus on the application of exosome treatment and diagnosis. Since 2017, more and more investors have joined the field of exosomes. Emerging exosome biotechnology companies have a surprisingly diverse set of strategies shaping the medical and commercial future of the emerging exosome field.

### 5.1 Codiak biosciences

Codiak Biosciences is a company engaged in the development of exosome drugs. Codiak has developed the engEx™ platform, which enables scientists to design and modify exosomes with different characteristics.Here, we will list three products.

#### 5.1.1 ExoIL-12

ExoIL-12 load IL-12 on the surface of exosomes and transport it to the tumor environment. At the same time, exoIL-12 can reduce the side effects caused by the distribution of IL-12 in the whole body. It has entered phase 1 clinical treatment in September 2020.

#### 5.1.2 ExoSTING™

ExoSTING™ is incorporating proprietary STING (stimulator of interferon genes) agonist inside the exosome to facilitate specific uptake in tumor-resident antigen-presenting cells. The high level of ptgfrn expression can deliver sting agonists to antigen-presenting cells (APCs) in the TME and induce the expression of interferon genes. Pre-clinical data studies suggest that inflammatory cytokine-driven adverse events is rare.

#### 5.1.3 ExoASO™-STAT6

Transcription factors such as STAT6 that regulate gene families are ideal targets for potent repolarizing of tumor-associated macrophages. ASO(antisense oligonucleotide, whose base sequence can be complementary to the specific target RNA sequence and affect the expression of the target gene, such as silencing it by binding to the corresponding mRNA) carrying a specific sequence on the surface of exosomes, and delivering it to highly immunosuppressive macrophages (M2). They designed exoaso-stat6 to make STAT6 ASO have the necessary transmission specificity to M2 macrophages. Their preclinical study of exoaso-stat6 showed that compared with free Aso, M2 polarized macrophages had preferential uptake, increased selective release, enhanced potency, and significantly reduced STAT6 mRNA, resulting in significantly reduced M2 gene expression and increased M1 gene expression.

### 5.2 Aegle therapeutics

Aegle Therapeutics, headquartered in Miami, Florida, uses extracellular vesicles, including exosomes, secreted by allogeneic bone marrow-derived mesenchymal stem cells to treat severe dermatological conditions, including dystrophic epidermolysis bullosa.

Aegle’s extracellular vesicles, contain and transport cargo, including proteins and genetic material. A recent publication entitled Dual mechanism of type VII collagen transfer by bone marrow mesenchymal stem cell extracellular vesicles to recessive dystrophic epidermolysis bullosa fibroblasts (Biochimie, 2018), Aegle researchers demonstrated that EVs transport and deliver type VII collagen protein as well as COL7A1 mRNA to diseased RDEB fibroblasts.

### 5.3 Evox therapeutics

Evox Therapeutics (Evox)developed a GMP-compliant, scalable, commercially viable manufacturing platform that forms part of the DeliverEX TM platform and assures the quality, safety, and efficacy of exosome therapeutics. Evox focuses on Inborn Errors of Metabolism (IEMs), which are genetic conditions that result from problems in metabolizing proteins, carbohydrates, fats, or other substances. Vox is developing a PAH-loaded exosome drug product. An exosome-loaded protein-based therapy is expected to be more effective than current therapeutic options. In addition, Evox is also evaluating approaches for delivering a long-acting nucleic acid payload as part of a pipeline.

## 6 Conclusion and prospects

To transform exosomes into a drug delivery system and ultimately successfully realize clinical applications, it is essential to improve the production efficiency of effective exosomes, load drugs into exosomes, and establish tumor organ targeting. First, obtaining exosomes efficiently is the basis for the industrial production of exosomes. We need tooptimize the production process to maximize the production and purification of exosomes and make them meet the needs of industrial production and clinical applications. Secondly, improving loading capacity is a crucial factor. The traditional drug loading method has low drug loading and will damage the drug or target cells. The improved exogenous methods will significantly improve the loading efficiency but may damage the exocytosis, and somatic vesicles may get cytotoxic, affecting further exosomal transport. Although many studies have successfully built a high-efficiency load transfer platform and controlled the sorting process to a certain extent, its scope of application is small, and some of it may have an impact on the activity of drug molecules. Finally, after we get high-yield and high-drug-loaded exosomes, the next step is to improve the targeting. In relevant studies on the surface modification of exosomes, researchers, by expressing specific targeting peptides, using physical methods and other techniques, have improved the targeted delivery efficiency of exosome-encapsulated drugs, demonstrating that exosomes as targeting carriers have great potential. If we wish to enter the stage of large-scale clinical application, many more targeted treatments should be explored.

The transformation of exosomes as a drug delivery system is a brand new and promising field for treating diseases, especially for the targeted therapy of solid tumors. We look forward to more extensive primary research and clinical application reports on the engineering modification of exosomes and targeted drug delivery to treat solid tumors.
